# Stabilized generation of human iPSC-derived liver organoids using a modified coating approach

**DOI:** 10.1093/biomethods/bpac034

**Published:** 2022-12-10

**Authors:** Yu Kamishibahara, Satoshi Okamoto, Takuya Ohkuma, Hideki Taniguchi

**Affiliations:** Department of Regenerative Medicine, Yokohama City University Graduate School of Medicine, Yokohama 236-0004, Japan; Department of Regenerative Medicine, Yokohama City University Graduate School of Medicine, Yokohama 236-0004, Japan; Division of Regenerative Medicine, Center for Stem Cell Biology and Regenerative Medicine, The Institute of Medical Science, the University of Tokyo, Tokyo 108-8639, Japan; Department of Regenerative Medicine, Yokohama City University Graduate School of Medicine, Yokohama 236-0004, Japan; Department of Regenerative Medicine, Yokohama City University Graduate School of Medicine, Yokohama 236-0004, Japan; Division of Regenerative Medicine, Center for Stem Cell Biology and Regenerative Medicine, The Institute of Medical Science, the University of Tokyo, Tokyo 108-8639, Japan

**Keywords:** coating, hiPSC, differentiation

## Abstract

Human-induced pluripotent stem cell (hiPSC)-derived hepatic cells are useful tools for regenerative medicine, and various culture substrates are currently used for their differentiation. We differentiated hiPSC-derived hepatic endoderm (HE), endothelial cells (ECs), and mesenchymal cells (MCs) using Laminin-511 (LN) coating to generate liver organoids, hiPSC-liver buds (hiPSC-LBs), which exhibited therapeutic effects when transplanted into disease model animals. Stably producing significant amounts of hiPSC-LBs is necessary for sufficient therapeutic effects. However, general precoating (standard coating) requires quick manipulation, often causing failure for inexperienced cell cultures, we thus tested direct LN addition to the culture medium (Direct coating). Using quantitative gene expression, flow cytometry, albumin secretion, and ammonia metabolism, we demonstrated that Standard and Direct coating similarly induce hiPSC-derived hepatocyte, mesodermal cell, EC, and MC differentiation. Standard and Direct coating-differentiated cells generated iPSC-LBs with equivalent hepatic functions. Furthermore, Direct coating enabled stable induction of differentiation independent of individual culture skills and reduced total amount of LN use as the same differentiated cell quality can be obtained upon LN supplementation at lower concentrations. In summary, the results of this study suggest that Direct coating could enable stable hiPSC-LB production at a low cost, thereby yielding mass cell production using hiPSCs.

## Introduction

Human-induced pluripotent stem cells (hiPSCs) are generated from somatic cells by expressing certain specific, well-defined genes [1]. hiPSCs exhibit infinite proliferation and differentiation capacity into all three germ layer-derived various cell types, including hepatocytes. Primary human hepatocytes are useful for drug screening, but they rarely proliferate and rapidly lose hepatic functions (such as drug-metabolizing capacity) *in vitro*, which limits their supply and use. Therefore, hiPSC-derived hepatic cells could provide a stable hepatocyte source for multiple applications [2, 3]. Several research groups have developed protocols for hiPSC-derived hepatocyte-like cell generation [4–6] for various modifications, such as treatment with small molecules [7, 8], co-culture with multiple cell types [9, 10], and use of various extracellular matrixes [11–13].

We have previously reported that three-dimensional (3D) cultures of several cells, including hepatic progenitor cells, using cell–cell interactions can create tissue structures that more closely resemble the liver [14, 15]. Liver organoids produced by this 3D culture method, that is hiPSC-liver buds (hiPSC-LBs), mimic the tissue structure of the liver with a vascular network between parenchymal cells and have improved liver functionality compared to flat 2D cultures [14]. Furthermore, we reported the production of hiPSC-LBs containing more than 1 × 10^8^ hepatocytes [16]. However, stable production of more than 1 × 10^8^ functional hepatocytes from hiPSCs requires highly skilled specialists. A common precoating method uses laminin-511 (LN), a cell adhesion substrate, by diluting it in phosphate-buffered saline (PBS), adding it to culture dishes, and incubating it until cell seeding (Standard coating) [17]. Standard coating involves a process of replacing the coating solution with the medium before cell seeding, which requires quick manipulation as the coating surface dries, potentially leading to failure. In addition, this approach is time-consuming for mass production. Recently, Miyazaki *et al*. [18] reported that undifferentiated hiPSC culture maintenance is possible by seeding cells with LNs mixed into the cell suspension (Uncoating). Although this method allows for omitting the coating solution replacement, we successfully attempted to differentiate hiPSCs into hepatocytes using this method.

In this study, we investigated whether LNs can be induced to differentiate by diluting LNs in medium instead of PBS and coating them, thereby eliminating coating solution replacement with the medium (henceforth termed as Direct coating). Direct coating allowed for iPSC to differentiate into not only hepatocytes but also mesodermal cells. In addition, functional iPSC-LBs could be successfully generated using cells differentiated by Direct coating. Direct coating made possible stable differentiation regardless of the skills of the specialists, and the required LN amount could be significantly reduced. These results suggest that Direct coating could enable hiPSC differentiation as well as Standard coating and reduce the risk of culture failure, thereby leading to stable hiPSC-LB production.

## Materials and methods

### Coating method for culture vessels

For the Standard coating method, 6-well flat-bottomed plates (BD Falcon) were coated with iMatrix-511 (kindly provided by Nippi) at a concentration of 0.26 µg/cm^2^ for 3 h at 37°C in a 5% CO_2_ incubator. iMatrix-511 was dissolved in PBS. After coating, remove the coating solution and replace it with a culture medium containing 10 µM Y-27632 (FUJIFILM Wako). hiPSCs were seeded immediately. For the Uncoating method, 0.26 µg/cm^2^ iMatrix-511 was added into the cell suspension containing 10 µM Y-27632 in 6-well plates without any coating. For the Direct coating method, a concentration of 0.26 µg/cm^2^ iMatrix-511 was added into the culture medium containing 10 µM Y-27632 for coating 6-well plates at 37°C in a 5% CO_2_ incubator right before cell seeding. hiPSCs were seeded in a medium.

### Human iPSC culture

hiPSC lines (Ff-I01s04) were provided by the Center for iPS Cell Research and Application, Kyoto University (CiRA). hiPSCs were maintained in StemFit AK02N (Ajinomoto) at 37°C in the presence of 5% CO_2_. Every 7 days at confluency, iPSCs were passaged by treatment with Accutase (Innovative Cell Technologies) for 5 min at room temperature. The cells were seeded onto culture vessels via the Uncoating method as described above. After passaging, the Y-27632-free culture medium was changed every 2 days. The use of hiPSCs in this study was approved by the Ethical Committee of Yokohama City University.

### Hepatic differentiation

The differentiation protocol for definitive endoderm (DE) cell, hepatic endoderm (HE) cell, and mature hepatocyte (MH) induction was described previously with modifications [16]. To derive DE cells, hiPSCs were seeded onto culture dishes with DE induction medium containing 10 µM Y-27632 either via the Standard, Direct, or Uncoating method as described above. The DE induction medium consisted of RPMI-1640 (Thermo Fisher Scientific) with 20% StemFit for Differentiation (Ajinomoto), which contained 33 ng/ml Activin A (Ajinomoto) and 2 µM CHIR99021 (Cayman). On the upcoming day, 500 µM sodium butyrate (Sigma) was added to the medium. On each of the following 2 days, CHIR99021 and sodium butyrate were omitted from the refreshed medium. To induce HE differentiation, DE cells were cultured for 8 days in an HE induction medium, consisting of Basic03 (Ajinomoto), which contained 1 mM L-Glutamine (Gibco), 1% nonessential amino acids (Gibco), 0.1 mM 2-mercaptoethanol (Thermo Fisher Scientific), and 1% Dimethyl sulfoxide (DMSO) (Nacalai Tesque). The medium was refreshed every day during the HE differentiation period. To differentiate MH cells, HE cells were cultured for 8 days in an MH induction medium consisting of Dulbecco's Modified Eagle Medium (DMEM) (Thermo Fisher Scientific) containing 5% fetal bovine serum (FBS) (MP Biomedicals), 100 nM Dexamethasone (Dex) (Sigma), and 20 ng/ml oncostatin M (OSM) (R&D). The medium was changed every 3 days.

### Endothelial cell and mesenchymal cell differentiation

The induction of endothelial cell (EC) and mesenchymal cell (MC) differentiation from iPSCs was based on our previous reports with modifications [16]. Briefly, for EC differentiation, hiPSCs were dissociated using Accutase and plated on culture dishes in StemFit AK02N containing 10 µM Y-27632 and iMatrix-511. The following day, the medium was replaced with a mesoderm induction medium consisting of DMEM/F12 (Thermo Fisher Scientific) with 20% StemFit for Differentiation, which contained 1% GlutaMAX, 25 ng/ml BMP4 (R&D), and 8 µM CHIR99021. After three more days, the mesoderm induction medium was replaced with an EC induction medium consisting of StemPro-34 SFM media (Thermo Fisher Scientific) supplemented with 200 ng/ml vascular endothelial growth factor (VEGF) (FUJIFILM Wako) and 2 µM forskolin (Cayman). The induction medium was refreshed every day. On Day 10 of differentiation, ECs were replated on culture dishes in an EC maintenance medium without Y-27632, but with iMatrix-511. The EC maintenance medium consisted of StemPro-34 SFM medium supplemented with 50 ng/ml VEGF. On the upcoming day, the medium was replaced with an EC expansion medium. The EC expansion medium was refreshed every 3 days. For MC differentiation, hiPSCs were dissociated using Accutase and plated on culture dishes in StemFit AK02N containing 10 µM Y-27632 and iMatrix-511. The following day, the medium was replaced with a mesoderm induction medium for 3 days, followed by 2 days of exposure to 10 ng/ml Platelet Derived Growth Factor (PDGF) BB (Peprotech) and 0.66 ng/ml Activin A. On Day 6 of differentiation, the medium was replaced with an MC induction medium consisting of DMEM/F12 with 20% StemFit for Differentiation, supplemented with 1% Glutamax, 10 ng/ml bFGF (FUJIFILM Wako), and 12 ng/ml BMP4. The MC induction medium was refreshed daily.

### Liver bud culture in microwells

To generate LBs from iPSCs, human iPSC-HEs/iPSC-ECs/iPSC-MCs (10:7:1 ratio) were resuspended in a mixture of the MH induction medium and KBM VEC-1 (KOHJIN BIO), then plated on the Elplasia microwell plates (Corning). Half of the medium was replaced with fresh medium every day. After 10 days, LBs were collected by gentle pipetting and used for *in vitro* maturity assessment. The LBs were evaluated using a Cell^3^iMager duos (SCREEN Holdings).

### Flow cytometry analysis

Cells were dissociated by treatment with 0.05% trypsin in ethylenediaminetetraacetic acid (EDTA) solution (Thermo Fisher Scientific) for several minutes at room temperature, then supplemented with DMEM/10% FBS. After centrifugation, the supernatant was discarded and the cells were washed with PBS. The cells were dispersed by pipetting, and 2 × 10^5^ cells were transferred to a 1.5-ml tube. We used the following antibodies for the flow cytometry (FCM) analysis: Alexa Fluor-488 anti-human Tra2-49 antibody (Biolegend), APC mouse anti-human CD184 (CXCR4) (BD), and anti-human CD133/1 (AC133) (Miltenyi Biotec). Data acquisition was performed using a BD FACSAria II instrument.

### Gene expression levels

Total RNA was isolated from iPSCs and iPSC-derived cells using PureLink^TM^ RNA Mini Kit (Thermo Fisher Scientific). cDNA was synthesized from 1 µg of total RNA with a High-Capacity cDNA Reverse Transcription Kit (Thermo Fisher Scientific). Quantitative PCR (qPCR) was performed with THUNDERBIRD^®^ Probe qPCR Mix (TOYOBO) and Universal Probe Library (Roche Life Science) using a LightCycler^®^ 480 II (Roche Life Science). The relative quantities of target mRNAs were determined using the 2^−ΔCt^ method. The values were normalized to 18S rRNA levels. The primer sequences used in this study are described in Supplementary Table S1.

### ELISA

Human iPSCs were differentiated into MHs for 20 days, and iPSC-LBs were cultured for 10 days. The culture supernatants, incubated for 24 h after fresh batch was added, were collected and analyzed to determine the albumin (ALB) secretion level by ELISA. The Human ALB ELISA Quantitation kit was purchased from Bethyl Laboratories and used according to the manufacturer’s instructions. The amount of ALB secreted was calculated using a standard curve.

### Ammonia metabolism and urea production assays

After addition of ammonium chloride, ammonia metabolism was evaluated by changing the ammonia concentration in the cell culture supernatant over a 24 h period. NH_4_Cl (2 mM) was added to the culture medium, and the medium was collected after 24 h for ammonium concentration measurement. The Ammonia Test Kit II (arkray) was used to measure ammonia clearance according to the manufacturer’s instructions. For measurement of urea production, the Quantichrom Urea Assay Kit (Bioassay systems) was used according to the manufacturer’s instructions.

### Statistical analysis

The data were calculated as the means ± standard error of the mean. Statistical significance was evaluated by the nonparametric Mann–Whitney U test. One-way ANOVA and Tukey’s post-hoc test were used to determine significant differences between more than two groups. *P *<* *0.05 were considered statistically significant.

## Results

### hiPSC-derived HE and MH generation using Direct coating

**Figure 1:**
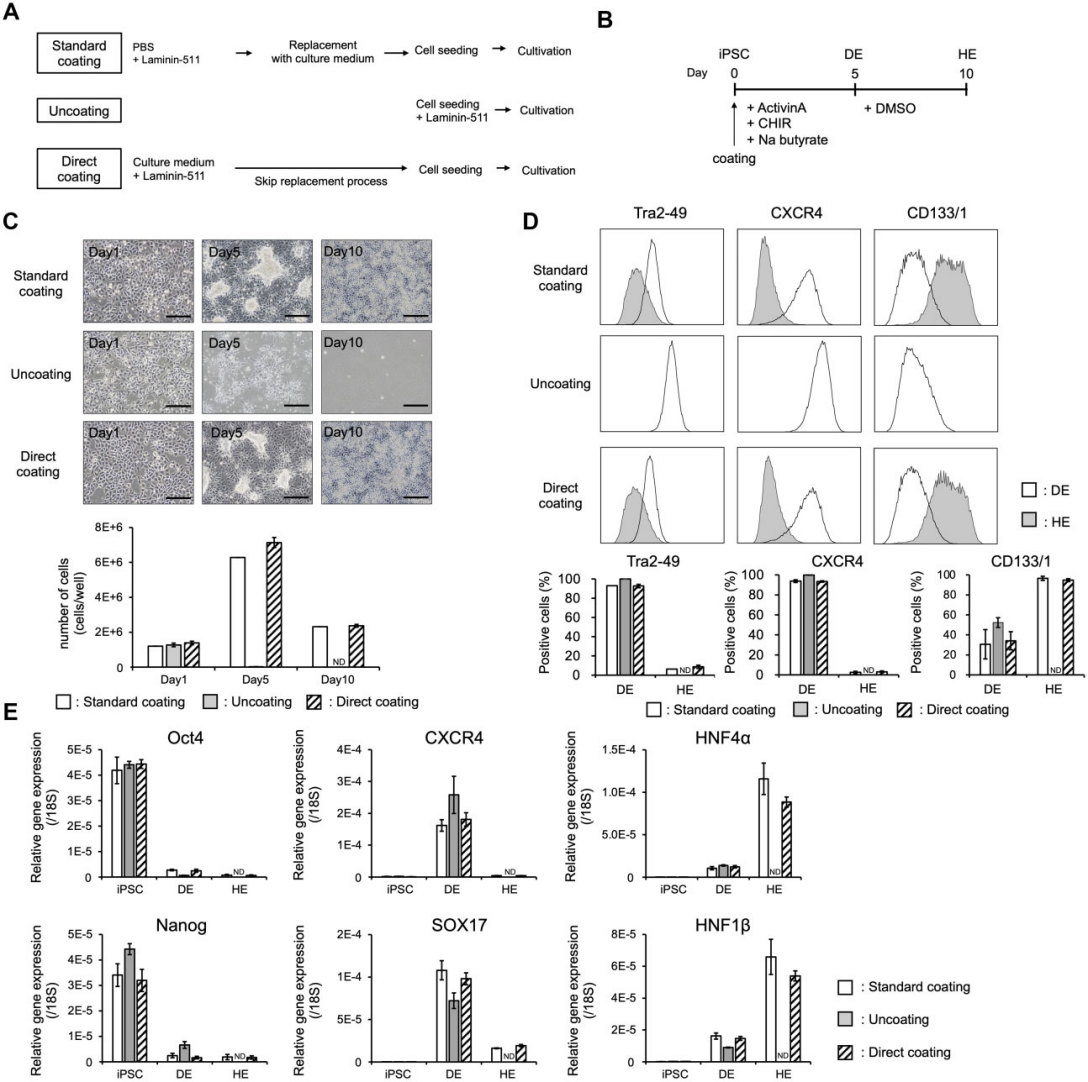
Differentiation of iPSC-derived HE via Direct coating. (A) Schematic drawing of the three coating methods. (B) Schematic representation of the DE and HE differentiation protocol. (C) (Upper) Morphology of iPSC-HEs differentiated using different coating methods between Days 1 and 10. (Lower) Number of cells using different coating methods. (D) FCM-based quantification of Tra2-49, CXCR4, and CD133/1. The data are represented as the means ± standard error of the mean (SEM), *n *=* *4. (E) Characterization of specific markers of each differentiation stage by qPCR analysis: pluripotency (*Oct4*, *Nanog*), DE (*CXCR4*, *SOX17*), and HE (*HNF4α*, *HNF1β*) markers. The data are represented as the means ± SEM, *n *=* *3. These results indicate that Uncoating could not allow for differentiating HEs from iPSCs, but Direct coating enables iPS-derived HE differentiation equivalent to Standard coating. Scale bar: 200 µm.

In this study, we examined three LN coating approaches: (i) a general precoating method in which LN is diluted in PBS (Standard coating); (ii) a method in which LN is mixed with cell seeding solution (Uncoating) as reported by Miyazaki *et al*.; and (iii) a method in which LN is precoated by diluting it with the cell culture medium (Direct coating) (Fig. 1A). To differentiate hiPSC-derived hepatocytes, we used a previously reported differentiation protocol with modifications (Fig. 1B). When hiPSCs were seeded via Standard coating, they differentiated into HE but not via Uncoating (Fig. 1C). We hypothesized that culturing these cells might be possible without the replacement of the medium by culturing with a coating medium instead of PBS (Direct coating). Rho-associated, coiled-coil containing protein kinase (ROCK) inhibitor Y-27632-containing medium was supplemented with LNs, poured into culture dishes, and incubated at 37°C right before cell seeding. Direct coating allowed differentiation into HEs as efficiently as Standard coating (Fig. 1C).

Next, we characterized hiPSC-derived HEs using FCM and qPCR analyses. The FCM analysis showed that the expression of undifferentiated and DE markers (Tra2-49 and CXCR4, respectively) decreased in HE (Day 10) cells compared with DE (Day 5) cells in the case of each coating method. However, the HE marker (CD133/1) expression tended to increase from DE to HE cells, except for the cells in the Uncoating culture (Fig. 1D). The qPCR gene expression analysis showed that the expression of undifferentiated markers (*Oct4* and *Nanog*) was the highest in iPSCs and decreased as the differentiation progressed from DE to HE cells (Fig. 1E). The expression of DE markers *CXCR4* and *SOX17* increased from iPSCs to DE cells and decreased as the differentiation progressed toward the HE. In addition, HE marker *HNF4α* and *HNF1β* expressions increased as differentiation progressed from iPSCs to HE cells (except for the Uncoating culture, Fig. 1E). Direct coating cultures displayed each differentiation-related gene expression peak at the same stage as Standard coating cultures with no significant difference in the expression levels between the peaks. These results indicate that hiPSC-derived HE differentiation was equivalent in the case of Direct and Standard coating.

**Figure 2:**
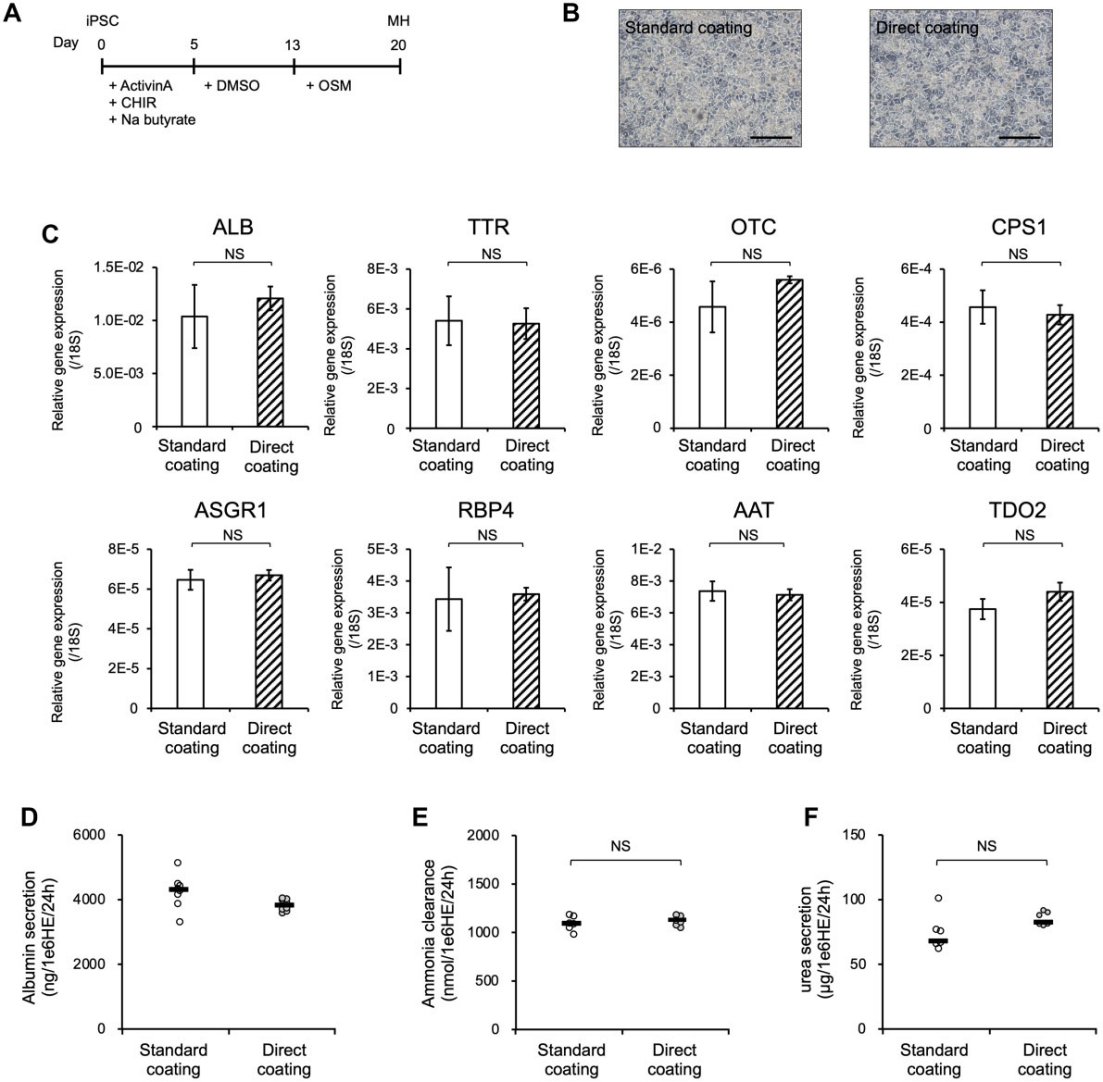
Differentiation of iPSC-derived MHs via a Direct coating. (A) Schematic representation of the MH differentiation protocol. (B) Morphology of iPSC-MHs differentiated using each coating method. (C) Gene expression analysis of the iPSC-MHs differentiated using each coating method. The data are represented as the means ± SEM, *n *=* *4. Differentiated MHs in the case of Direct coating were not significantly different from those of Standard coating [(ALB): *P *=* *0.79; (TTR): *P *=* *0.78; (OTC): *P *=* *0.49; (CPS1): *P *=* *0.84; (ASGR1): *P *=* *0.73; (RBP4): *P *=* *0.93; (AAT): *P *=* *0.82; (TDO2): *P *=* *0.58]. (D) ALB secretion of iPSC-MHs at Day 20, determined by ELISA. The data are represented as the means ± SEM, *n *=* *8. (E) Ammonia metabolism of iPSC-MHs at Day 20. The data are represented as the means ± SEM, *n *=* *6. (F) Urea secretion of iPSC-MHs at Day 20. The data are represented as the means ± SEM, *n *=* *6. These results indicate that Direct coating enabled iPSC-derived MH differentiation equivalent to Standard coating. Scale bar: 200 µm.

We next assessed the hiPSC-derived MHs following differentiation via Direct coating. We performed morphological comparisons, characterization, and functional analysis of hiPSCs-MHs in Standard and Direct coating cultures (Fig. 2A). No difference in MH morphology could be observed between the coating methods (Fig. 2B). The expression levels of MHs markers were comparable between Standard and Direct coating samples with no significant difference (Fig. 2C). In addition, hiPSC-MHs produced high levels of ALB (over 3000 ng/1 × 10^6^ HE cells/24 h) (Fig. 2D). Furthermore, they not only exhibited ammonia clearance capacity (over 1000 nmol/1 × 10^6^ HE cells/24 h) but also urea secretion activity (over 80 µg/1 × 10^6^ HE cells/24 h) (Fig. 2E and F). These results suggest that MHs could be successfully differentiated from hiPSCs using Direct coating.

### hiPSC-derived endothelial and MC generation via Direct coating

**Figure 3:**
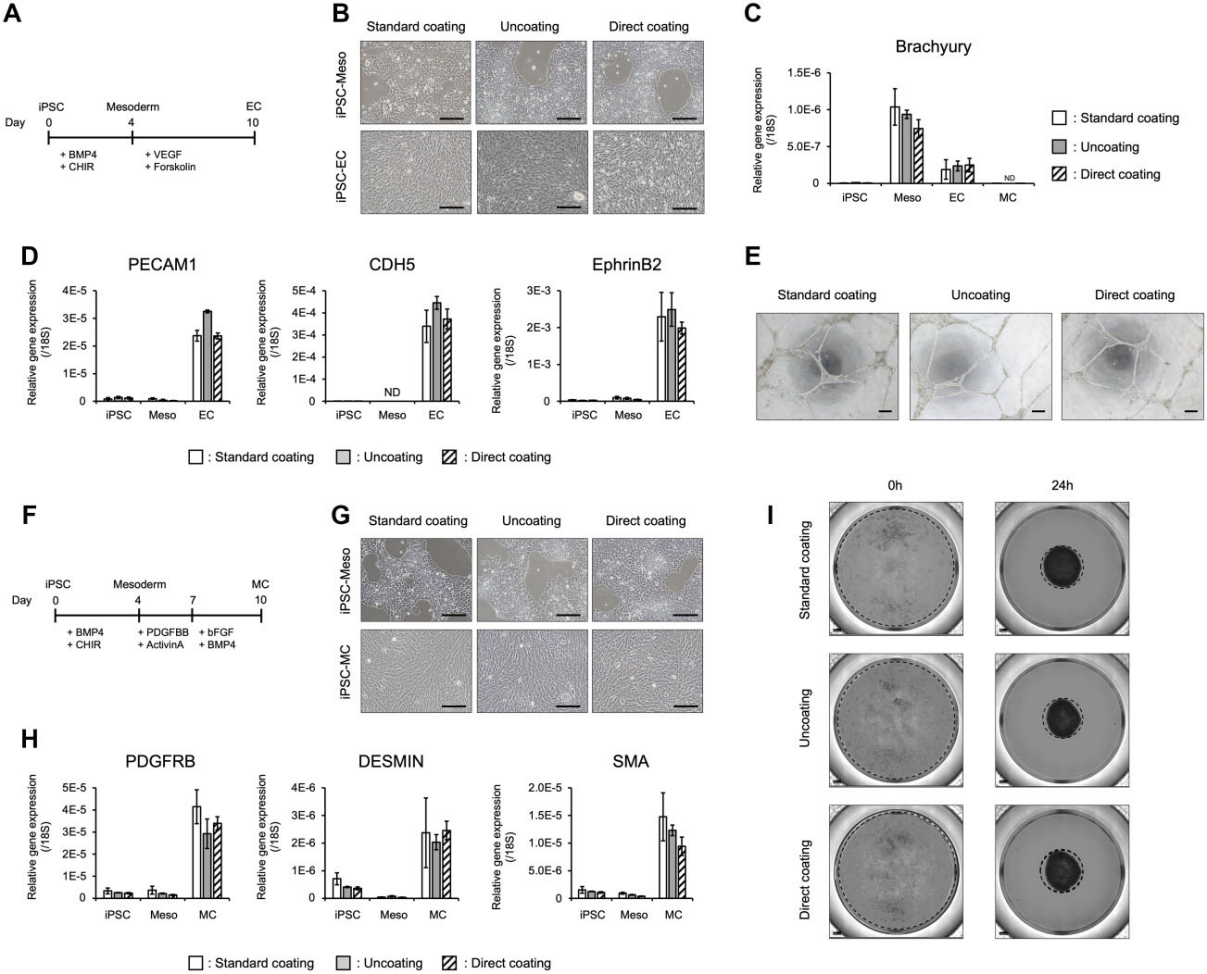
Differentiation of iPSC-derived ECs and MCs via Direct coating. (A) Schematic representation of the EC differentiation protocol. (B) Morphology of iPSC-ECs differentiated using different coating methods. (C) Gene expression analysis of the mesoderm cell marker *Brachyury*. Meso, iPSC-derived mesoderm on Day 4; EC, iPSC-derived EC on Day 10; MC, iPSC-derived MC on Day 10. The data are represented as the means ± SEM, *n *=* *4. (D) Gene expression analysis of EC markers *PECAM1*, *CDH5*, and *EphrinB2*. The data are represented as the means ± SEM, *n *=* *4. (E) Endothelial tube formation assay on Matrigel. (F) Schematic representation of the MC differentiation protocol. (G) Morphology of iPSC-MCs differentiated using different coating methods. (H) Gene expression analysis of MC markers *PDGFRB*, *DESMIN*, and *SMA*. The data are represented as the means ± SEM, *n *=* *4. (I) Contraction assay by iPSC-MC. These results indicate that both Direct and Standard coating enabled iPSC-derived EC and MC differentiation. Scale bar: 200 µm.

As described above, Direct coating successfully allowed iPSC differentiation into HE cells at an equivalent level to Standard coating. We investigated whether EC and MC differentiation could be achieved by Direct coating. To reproducibly generate ECs from hiPSCs, we modified our protocol from previous reports (Fig. 3A) [17]. The hiPSC-derived mesodermal cells and ECs from Standard coating, Uncoating, and Direct coating cultures showed no difference in cell morphology (Fig. 3B). Comparing the mesoderm marker *Brachyury* expression at each differentiation stage, increasing expression could be observed from iPSCs to mesoderm regardless of which coating method was used, and expression reduction was detected with progressing differentiation from ECs and MCs (Fig. 3C). EC marker gene (e.g., *PECAM1*, *CDH5*, and *EphrinB2*) expression increased as differentiation progressed from hiPSCs to ECs (Fig. 3D). In addition, differentiated iPSC-ECs exhibited endothelial potential, such as tube formation ability (Fig. 3E).

Finally, we challenged hiPSC-derived MCs differentiated via Direct coating using a protocol modified from previous reports (Fig. 3F) [16]. hiPSC-derived MCs from the three coating cultures showed no difference in cell morphology (Fig. 3G). In the case of each coating method, MC marker gene (e.g. *PDGFRB*, *DESMIN*, and *SMA*) expression increased as the differentiation progressed from hiPSCs to ECs (Fig. 3H). In addition, when hiPSC-MC-containing cells were seeded on Matrigel, they showed strong contractile activity (Fig. 3I). These results indicate that Direct coating enables the differentiation of not only hiPSC-derived ECs but also MCs. Interestingly, both ECs and MCs could be differentiated using Uncoating.

### Liver organoid generation from human iPSCs

**Figure 4:**
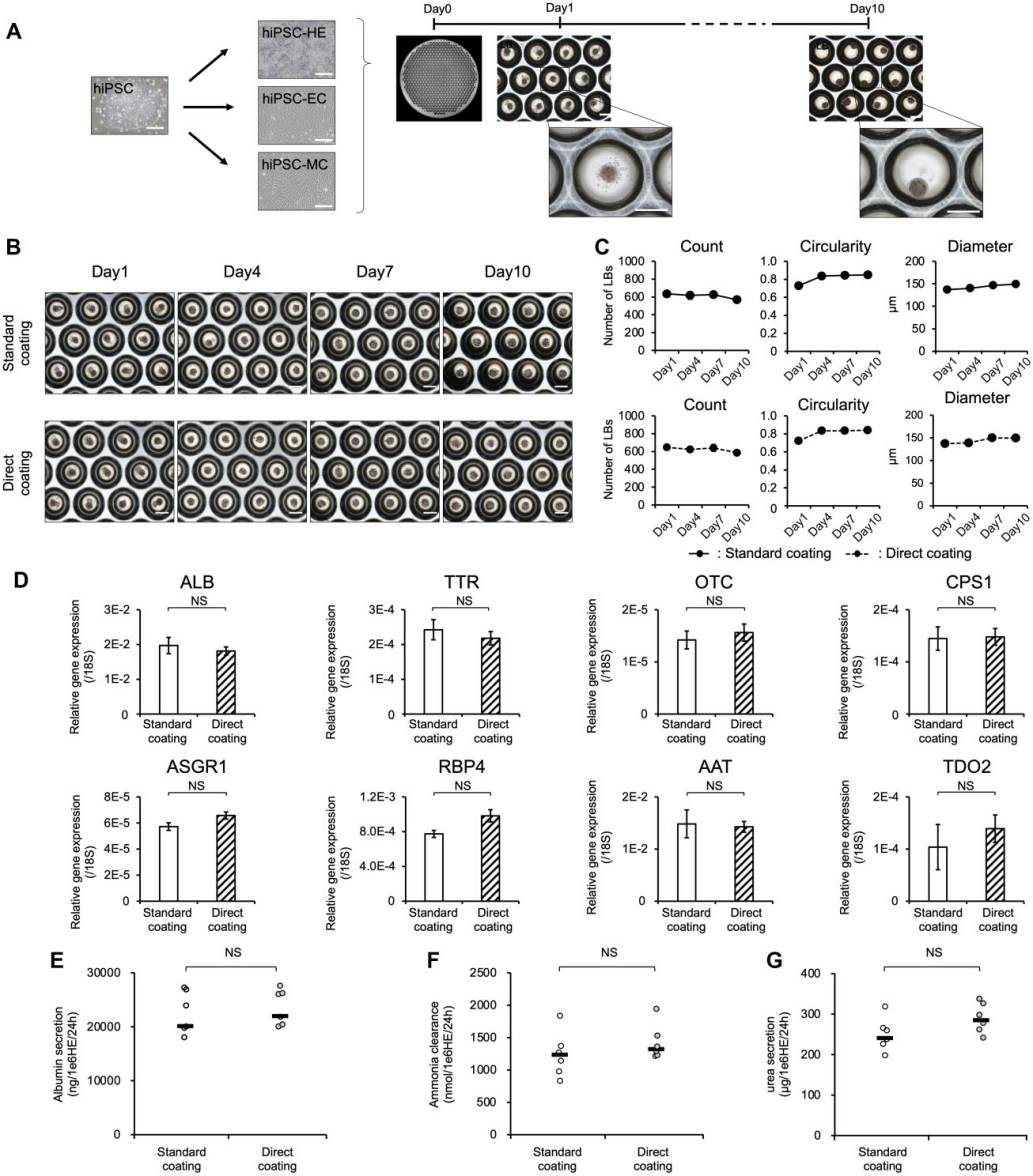
Generation of iPSC-derived liver organoids via Direct coating. (A) Overview of the protocol used for iPSC-derived LB generation. (B) Morphology of iPSC-LBs differentiated using different coating methods. (C) Morphological analysis of the LBs generated by cells differentiated using each coating method. The data are represented as the means ± SEM, *n *=* *6. (D) qPCR analysis of various marker genes for hepatic cells in iPSC-LBs. The data are represented as the means ± SEM, *n *=* *3. (E) ALB secretion of iPSC-LBs on Day 10, determined by ELISA. The data are represented as the means ± SEM, *n *=* *7. (F) Ammonia metabolism of iPSC-LBs on Day 10. The data are represented as the means ± SEM, *n *=* *7. (G) Urea secretion of iPSC-LBs on Day 10. The data are represented as the means ± SEM, *n *=* *7. These results indicate that the LBs generated using Direct and Standard coating showed the same functions. Scale bar: 200 µm.

In previous reports, we showed that functional liver organoids (LBs) can be generated from hiPSCs [14–16]. LBs consist of three progenitors or immature cells: HEs, ECs, and MCs (Fig. 4A). Therefore, HEs, ECs, and MCs were differentiated from hiPSCs via Direct coating, and LBs were prepared by mixing and culturing them. To characterize iPSC-LBs, we performed qPCR analysis and several hepatic functional analyses.

The generated LBs underwent image analysis using a cell scanner to quantify their morphological information (Fig. 4B). hiPSC-LBs generated via Direct coating exhibited high circularity and uniform size similar to those of Standard coating cultures (Fig. 4C). The gene expression levels of mature hepatic cell markers were comparable between hiPSC-LBs generated via Direct and Standard coating with no significant difference between them (Fig. 4D). In addition, iPSC-LBs exhibited high ALB production (over 20 000 ng/1 × 10^6^ HE cells/24 h) (Fig. 4E). These cells not only displayed ammonia clearance capacity (over 1000 nmol/1 × 10^6^ HE cells/24 h) but also urea secretion ability (over 200 µg/1 × 10^6^ HE cells/24 h) (Fig. 4F and G). These results suggest that hiPSC-derived HE differentiated from using the Direct coating could be used to generate LBs with highly functional hepatocytes.

### The advantages of Direct coating

**Figure 5:**
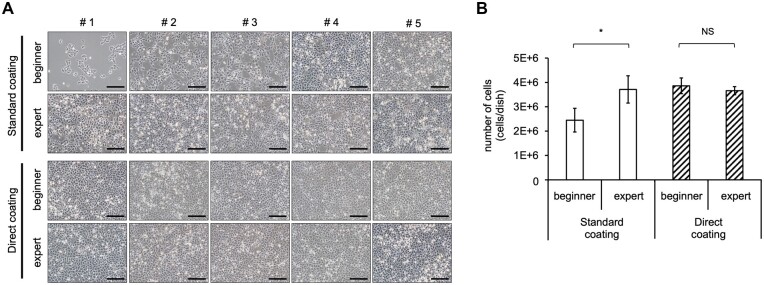
Direct coating enables stable culture independent of culture handling skills. (A) Morphology of cells seeded by beginners and experts. The images show the aspects of cell adhesion at 24 h after seeding. (B) Total number of cells under each condition. The data are represented as the means ± SEM, *n *=* *5, *P <* *0.05. This result indicates that even beginners are less likely to fail by using the Direct coating. Scale bar: 200 µm.

The above-described results demonstrated that Direct coating enables iPSC-derived HE cell, EC, and MC differentiation and functional iPSC-LB generation. Hence, we investigated the advantages of Direct coating. First, coating time was examined. Both coating methods required at least 15 min of incubation (Supplementary Fig. S1). Next, we examined whether cell culture handling would be different for beginners and experts using each coating method. Standard coating involves a replacement process. If beginners perform it, the culture vessel surface dries and optimal culture cannot be performed. In contrast, Direct coating enables optimal culture handling as this replacement process step can be omitted (Fig. 5A and B). However, all experts were able to culture by either method.

**Figure 6:**
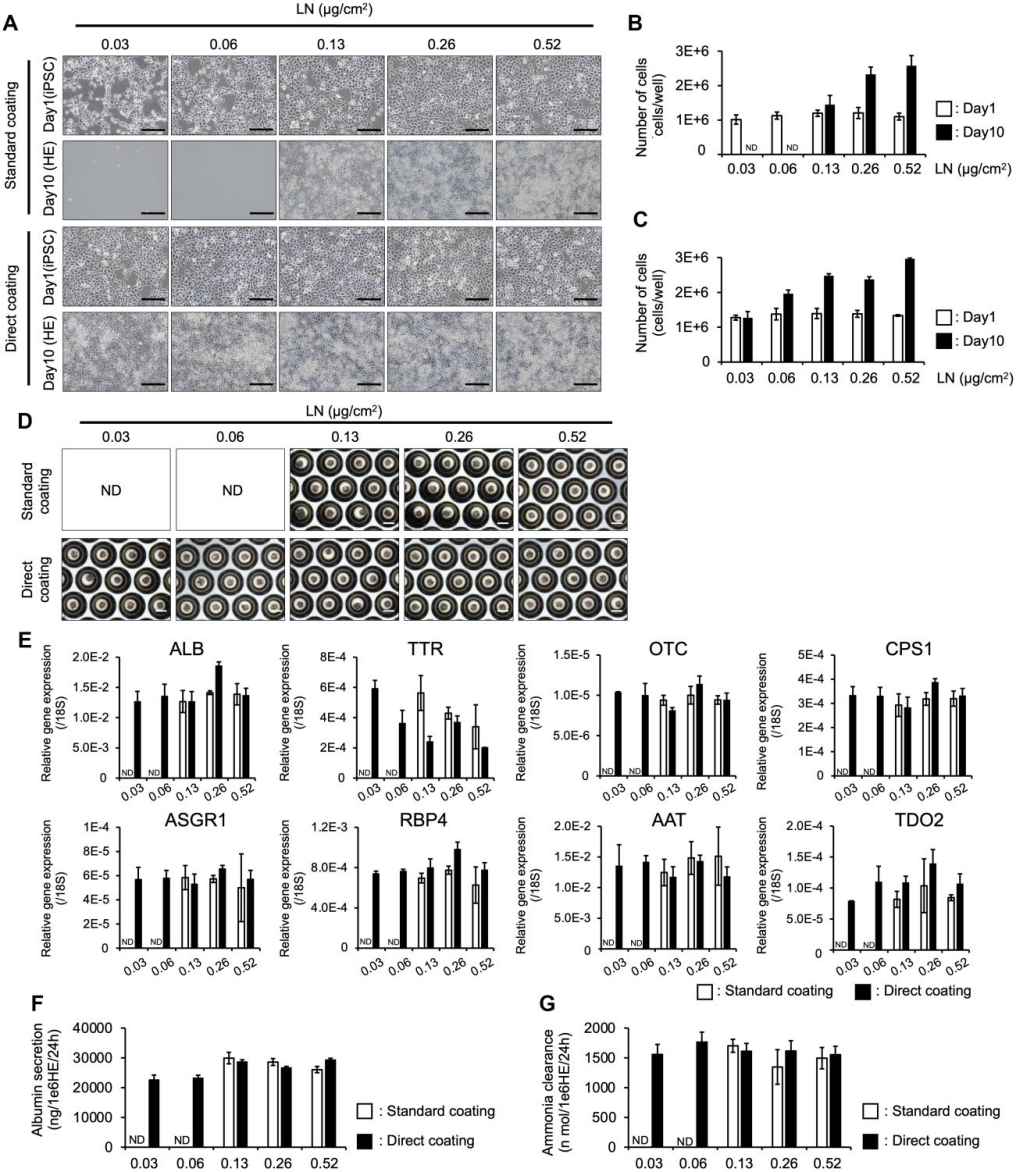
Direct coating enables laminin concentration reduction. (A) Morphology of iPSC-HEs at each laminin concentration on Days 1 and 10. (B) Number of cells at each concentration using Standard coating. The data are represented as the means ± SEM, *n *=* *3. (C) Number of cells at each concentration using Direct coating. The data are represented as the means ± SEM, *n *=* *3. (D) Morphology of iPSC-LBs at each laminin concentration on Day 10. (E) qPCR analysis of various marker genes for mature hepatic cells in iPSC-LBs. The data are represented as the means ± SEM, *n *=* *3. (E) ALB secretion of iPSC-LBs on Day 10, determined by ELISA. The data are represented as the means ± SEM, *n *=* *3. (G) Ammonia metabolism of iPSC-LBs on Day 10. The data are represented as the means ± SEM, *n *=* *3. These results indicate that Direct coating allows for culture conditions with a reduced laminin concentration. Scale bar: 200 µm.

In the study of Miyazaki *et al*., Uncoating could be used to reduce the amount of LN and culture-undifferentiated hiPSCs. Therefore, we investigated whether Direct coating would allow HE differentiation by reducing the amount of LN. The Standard coating could be reduced from the manufacturer’s recommended concentration of LN (0.52 µg/cm^2^) to 1/4 amount (0.13 µg/cm^2^). However, Direct coating reduced the number of cells recovered on Day 10, though differentiation was possible even if the amount was reduced until 1/16 (0.03 µg/cm^2^) (Fig. 6A–C) but not lower (Supplementary Fig. S2). Next, we generated and evaluated LBs using HEs differentiated at each LN dose (Fig. 6D). The gene expression levels of MH markers were similar among the LN doses for all genes with no significant differences in ALB production or ammonia metabolism (Fig. 6E–G). These results suggested that Direct coating could enable stable LB generation even at reduced LN levels.

## Discussion

In this study, we showed that our new precoating method, Direct coating, allows the omission of PBS replacement with the culture medium, an essential step in Standard coating, and the induction of hiPSC-derived hepatocyte and mesoderm cell differentiation similar to Standard coating. In addition, Direct coating can not only stabilize individual culture skills but also reduce the amount of LN during hiPSC differentiation.

The culture substrates used for hiPSC differentiation into multiple cell types vary widely [19–23], but generally, they must be pre-applied to the culture vessels. Culture vessels coated with substrates such as LN cannot be stored, they thus need to be prepared in time. Miyazaki *et al*. reported that undifferentiated hiPSCs can be cultured by LN supplementation into the cell suspension (Uncoating) [18]. However, differentiation failed when we attempted hiPSC-hepatic cell lineage induction via Uncoating. Therefore, we investigated the possibility of diluting and coating LNs with the medium instead of PBS and observed successful hiPSC differentiation into hepatocytes (Figs 1 and 2). Reimhult *et al*. [24] reported that serum ALB and other substances in the culture medium block the surface of the culture vessel, thereby inhibiting nonspecific protein binding to the plastic surface. The use of PBS for culture substrate dilution is usually considered to be based on the same empirical principle. We thus believed that Direct coating might not allow differentiation as LNs would be prevented from attaching to the culture surface. Interestingly, when the experiment was performed, iPSC differentiation was possible.

In order to generate hiPSC-LBs, ECs and MCs were differentiated from hiPSCs via Direct coating (Fig. 3). We generated LBs by mixing with iPSC-derived HE cells, ECs, and MCs via the Direct coating (Fig. 4). Multiple researchers, including us, previously reported that co-culture with ECs improved hepatocyte maturation [16, 25–27]. In fact, LBs generated with cells differentiated by Direct coating showed several-fold higher values than those in 2D culture for hepatic functions, such as ALB secretion, suggesting that the hepatocytes were more mature. Furthermore, Direct coating reduced the risk of handling-related failure and the amount of LN used during differentiation, suggesting the possibility of stabilizing cell production and reducing culture cost.

The modified coating method in this study was performed using the E8 fragment of LN-511, which is widely used in hiPSC research [17]. However, different culture substrates are used to differentiate hiPSCs into various cell types, and it remains to be determined whether Direct coating is effective for substrates other than LN. If the same method could be applied to other culture substrates, it might lead to the stabilization of various cellular production processes, and further studies are promoted.

In conclusion, we have developed Direct coating, a new coating method for hiPSC differentiation, enabling it to stabilize hiPSC differentiation independent of individual culture handling skills and achieve more stable hiPSC-LB production than ever before.

## Supplementary Material

bpac034_Supplementary_Data

## Data Availability

The data generated during and/or analyzed during the current study are available from the corresponding author on reasonable request.
