# Supplementary material for: Stabilized generation of human iPSC-derived liver organoids using a modified coating approach
Source: Biol Methods Protoc. 2022 Dec 10;8(1):bpac034. doi: 10.1093/biomethods/bpac034 (PMC9869720; doi:10.1093/biomethods/bpac034)
Supplement: bpac034_Supplementary_Data [file bpac034_supplementary_data.pdf]

Supplemental information

Stabilized generation of human iPSC-derived liver organoids using a modified coating approach

Yu Kamishibahara[1], Satoshi Okamoto[1,2,*], Takuya Ohkuma[1], and Hideki Taniguchi[1,2,*]

[1]Department of Regenerative Medicine, Yokohama City University Graduate School of Medicine, 3-9 Fukuura, Kanazawa-ku, Yokohama 236-0004, Japan

[2]Division of Regenerative Medicine, Center for Stem Cell Biology and Regenerative Medicine, The Institute of Medical Science, the University of Tokyo, 4-6-1 Shirokanedai, Minato-ku, Tokyo 108-8639, Japan

[*]Corresponding authors

E-mail: rtanigu@ims.u-tokyo.ac.jp (H.T.)

sokamoto@yokohama-cu.ac.jp (S.O.)

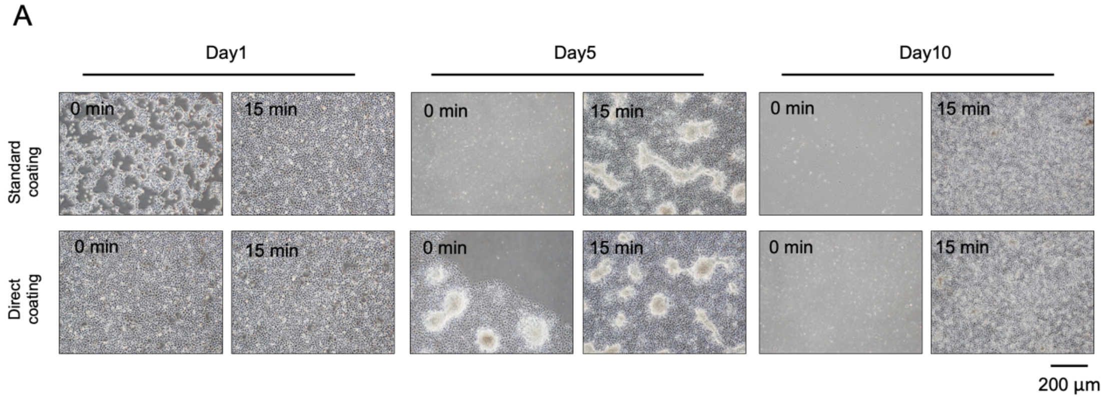

Supplementary Fig. S1. Examination of coating time.

(A) Morphology of iPSC-HEs due to the difference in coating time depending on each coating method after 24h adhesion. Scale bar: 200 μm.

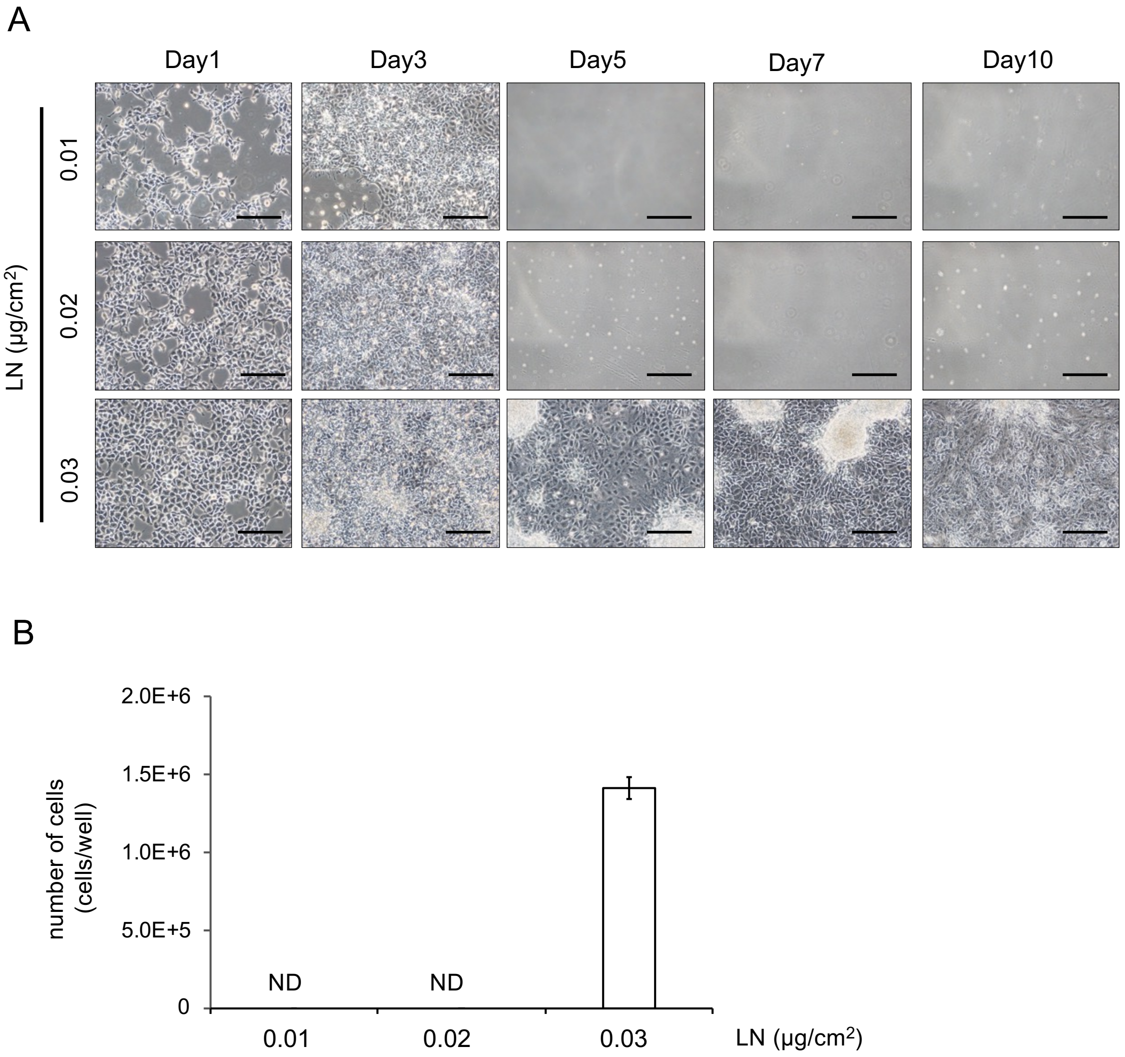

Supplementary Fig. S2. Differentiation of iPSC-HEs at low LN concentrations (Direct coating).

(A) Induction of iPSC-HE differentiation at low LN concentrations.

(B) Number of cells on iPSC-HE at low LN concentrations.

Supplementary Table 1 Primers used for qPCR

Gene	Forward Primer	Reverse Primer
OCT4	CTTCGCAAGCCCTCATTTC	GAGAAGGCGAAATCCGAAG
NANOG	ATGCCTCACACGGAGACTGT	CAGGGCTGTCCTGAATAAGC
CXCR4	ACGCCGAGTTGAGCAAGA	TCTGCCTCCTCCACGAAG
SOX17	ATTGGGATCAGCATCGACTC	CAAACTCACACCCTTGCTTG
HNF4α	TCAGACCCTGAGCCACCT	AGCAACGGACAGATGTGTGA
HNF1β	CCTCTCACCTGATGGTAAAATGA	GGATATTCGTCAAGGTGCTGA
AFP	TGTACTGCAGAGATAAGTTTAGCTGAC	TCCTTGTAAGTGGCTTCTTGAAC
ALB	AATGTTGCCAAGCTGCTGA	CTTCCCTTCATCCCGAAGTT
TTR	GCCGTGCATGTGTTCAGA	GCTCTCCAGACTCACTGGTTTT
OTC	CACCTTCAGGCAGCTACTCC	TGGTACCATTCTCTTTGGCATA
CPS1	CAAGTTTTGCAGTGGAATCG	ACTGGGTAGCCAATGGTGTC
AAT	CCATCTTCTTCCTGCCTGAT	GGTAAATGTAAGCTGGCAGACC
RBP4	CCAGAAGCGCAGAAGATTG	TTTCTTTCTGATCTGCCATCG
ASGR1	GCTGGAGAAACAGCAGAAGG	CGCAGGTCAGACACGAACT
TDO2	AAGAGGAATTCATAAGGATTCAGG	AGCACCTCTTTTTGCTTCTGA
TAT	CCATGATTTCCCTGTCCATT	GGATGGGGCCATAGCCATTAT
Brachyury	GCTGTGACAGGTACCCAACC	CATGCAGGTGAGTTGTCAGAA
PECAM1	CAGAGAGACCGGCTGTGG	CATTGTTCCCGGTTTCCA
CDH5	GCAGTCCAACGGAACAGAA	CATGAGCCTCTGCATCTTCC
EphrinB2	TCTTTGGAGGGCCTGGAT	GATCCAGCAGAACTTGCATCT
PDGFRB	CATCTGCAAAACCACCATTG	GAGACGTTGATGGATGACACC
DESMIN	ACAACCTGCTCGACGACCT	TTCTCTGCTTCTTCCTTCAACTG
SMA	CCTATCCCCGGGACTAAGAC	TTGTCACACACCAAGGCAGT
